# The pattern, change and driven factors of vegetation cover in the Qin Mountains region

**DOI:** 10.1038/s41598-020-75845-5

**Published:** 2020-11-25

**Authors:** Chenlu Huang, Qinke Yang, Yuhan Guo, Yongqiang Zhang, Linan Guo

**Affiliations:** 1 College of Urban and Environment, Northwest University, Xi’an, 710127 China; 2 Key Laboratory of Water Cycle and Related Land Surface Processes, Institute of Geographic Sciences and Natural Resources Research, The Chinese Academy of Sciences, Beijing, 100101 China; 3 Aerospace Information Research Institute, Chinese Academy of Sciences, Beijing, 100101 China

**Keywords:** Environmental impact, Climate-change impacts

## Abstract

The Qin Mountains region is one of the most important climatic boundaries that divide the North and South of China. This study investigates vegetation covers changes across the Qin Mountains region over the past three decades based on the Landsat-derived Normalized Difference Vegetation Index (NDVI), which were extracted from the Google Earth Engine (GEE). Our results show that the NDVI across the Qin Mountains have increased from 0.624 to 0.776 with annual change rates of 0.0053/a over the past 32 years. Besides, its abrupt point occurred in 2006 and the change rates after this point increased by 0.0094/a (R^2^ = 0.8159, p < 0.01) (2006–2018), which is higher than that in 1987–1999 and 1999–2006. The mean NDVI have changed in different elevation ranges. The NDVI in the areas below 3300 m increased, such increased is especially most obviously in the cropland. Most of the forest and grassland locate above 3300 m with higher increased rate. Before 2006, the temperature and reference evapotranspiration (PET) were the important driven factors of NDVI change below 3300 m. After afforestation, human activities become important factors that influenced NDVI changes in the low elevation area, but hydro-climatic factors still play an important role in NDVI increase in the higher elevations area.

## Introduction

Vegetation is usually regarded as an important indicator which can influence biodiversity and ecological process as well as an essential medium in water and energy balance, carbon cycling and so on^1–3^. Over the past several decades, the vegetation cover has experienced a notable change both at regional and continental scales under the global changing^4^. Hence, comprehensive studies of long-term vegetation cover change have become a hot issue, which is important for a better understanding of the spatial and temporal variation in the terrestrial ecosystem and is, thus, beneficial to regulate regional ecosystem balance under environment changes in the past century^5^. Observations based on remote sensing provided a chance for monitoring vegetation cover dynamics, more frequently, more accurately both at various spatial and temporal scales^6^. The Normalized Difference Vegetation Index (NDVI) is the most widely used remote sensing spectral index to track the vegetation cover change around the world^7^. For example, Du claimed that vegetation activity in Xinjiang showed an overall increasing tendency over the past three decades during the spring, autumn and summer seasons based on the Global Inventory Monitoring and Modeling System (GIMMS) NDVI dataset from 1982 to 2012^2^. Zhao discovered a significant increase of annual and seasonal NDVI from 2000 to 2014. He also claimed that “Grain for Green Project” (GFGP) had an important influence on vegetation dynamics. There is a strong correlations between the cumulative afforestation area and annual NDVI in Yan’an and Yulin from 2000 to 2013 based on the Moderate Resolution Imaging Spectroradiometer (MODIS) NDVI dataset^8^. Zhang combined the GIMMS with MODIS to quantify the response of vegetation to climate change and found that NDVI show an upward trend before the mid- or late 1990s mainly due to the fact that the climate tends to be hotter and drier under the effects of drought^9^. Deng analyzed the distribution and change trend of NDVI in the narrow Qin Mountains (the narrow here means the part of Qin Mountains within Shaanxi Province in China) based on the MODIS dataset, the results showed that the vegetation cover in narrow Qin Mountains increased significantly from 2000 to 2015, besides, the effect of the influence factor on the change of NDVI in the Qin Mountains is precipitation > temperature > Potential Evapotranspiration^10^. However, these researches still have some insufficiencies because most of them were based on the NDVI derived from GIMMS dataset, which have a long-term available dataset but were conducted only at the coarse resolution level. This lack of data might lead to the loss of vital spatial information at the regional scale. Besides, NDVI derived from MODIS dataset has a relatively high resolution, but unfortunately it cannot be used to monitor the vegetation change before 2000, and the processes for Landsat satellite images with more detail information in the large region needs a high requirement of computer capacity and more time. Fortunately, the Google Earth Engine (GEE) platform, a cloud-based large-scale computational facility for processing and analyzing large geospatial datasets^11^, provides a high-efficiency platform to monitor the vegetation cover change based on the high-resolution images^12^. Robinson calculated the NDVI by using Landsat surface reflectance dataset based on the GEE and compare the Landsat-derived NDVI with the MODIS-derived NDVI, which provided an ideal technical guidance in the dynamic analysis of vegetation cover^7^. Additionally, the topology is also important especially in the regions like the Qin Mountains that have the complex topology; however, lots of researches just analyzed the distribution and change trend of NDVI and the relationship between NDVI and climate factors, but the topology was neglected.

As the geographical line dividing multiple historical special significance and the sensitive zone in China, the Qinling, or Qin Mountains, have experienced complex and significant changes in vegetation cover in the past century due to climate change and strong human activities^10^. So, this study uses long-time series of Landsat-derived imagery at 30 m resolution based on the GEE platform to detect the pattern and change across the Qin Mountains region and investigates major driven factors of vegetation cover change. Thus, questions of this study tries to answer include: (1) when and where have vegetation changes occurs over the past three decades in the Qin Mountains region? and (2) What are the major reasons causing NDVI changes over the past decades in this area?

## Results

### The spatial distribution and change of annual average NDVI

**Figure 1:**
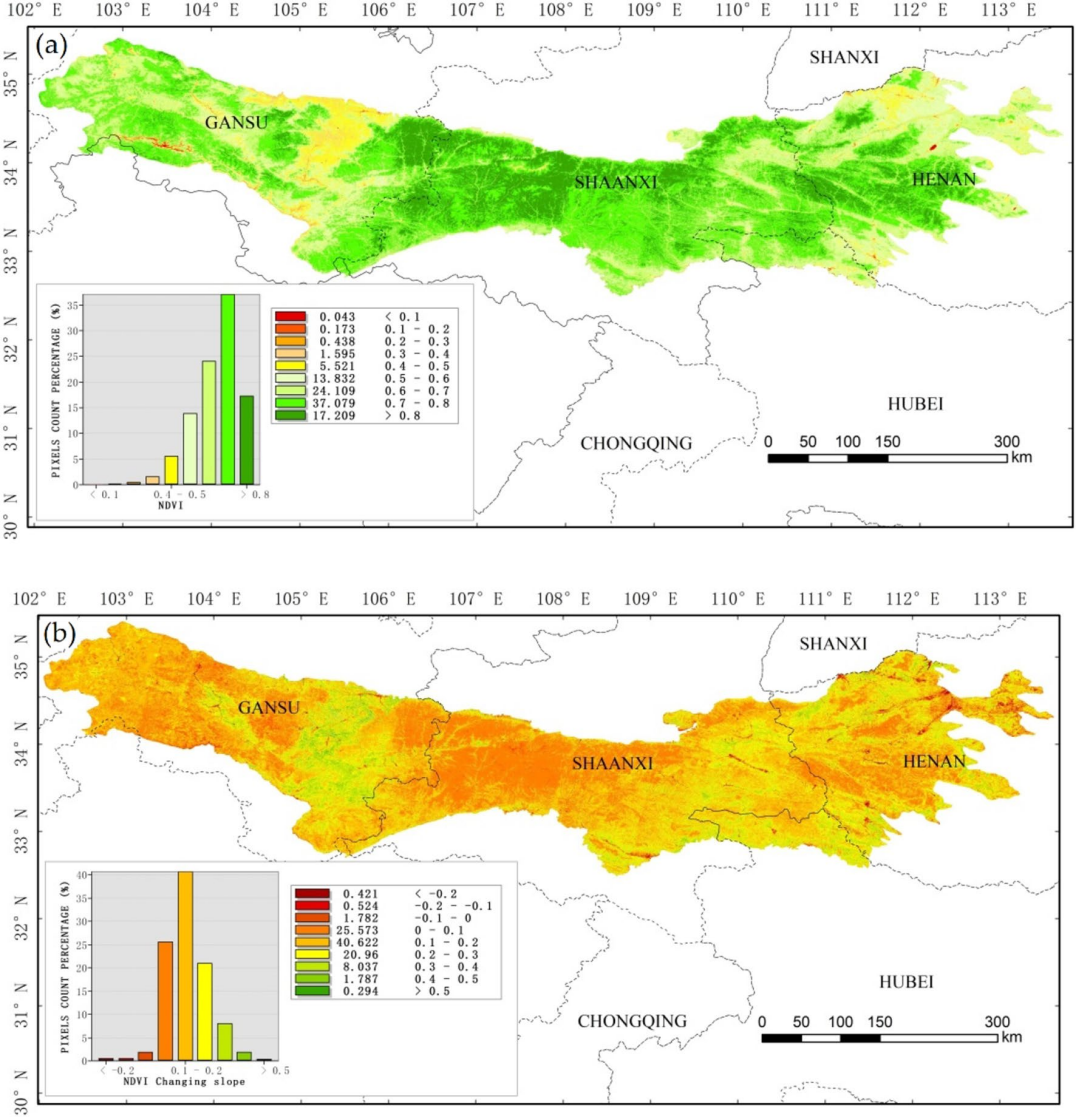
Spatial distribution (**a**) and changing trend (**b**) of NDVI in Qin Mountains during 1987–2018. The Landsat-derived NDVI here calculated from the Google Earth Engine (GEE) with high resolution and long time series. Theil–Sen Trend method that available in the GEE platform used to observe the changing trend of NDVI. The final map created in the ArcGIS 10.2.

Figure 1 shows that the spatial distribution and changing trend of NDVI from 1987 to 2018 in the Qin Mountains. Figure 1a shows that the spatial distribution of NDVI has a high degree of heterogeneity across the Qin Mountains. There are 92.2% of the Qin Mountains areas with high NDVI, i.e. NDVI > 0.5. Among these areas, the NDVI in the middle of Shaanxi and Henan are reach up to 0.8. There are less than 10% of the total areas with low NDVI, which are mainly located in the west and middle of Gansu province and south and north of Henan province.

It can be seen clearly from Fig. 1b that the increased slope is mainly concentrated on the range of 0–0.3, according for 87.38% of the total areas. The regions with lower NDVI show a rapidly increasing trend from 1987–2018, the slope in those area higher than 0.2. However, the NDVI in most areas such as middle of Shaanxi and Henan and the west of Gansu Province have a higher NDVI value and increased slowly (the changing slope less than 0.2) from 1987 to 2018.

### NDVI changing trend (1987–2018)

#### NDVI changes at regional scale

**Figure 2:**
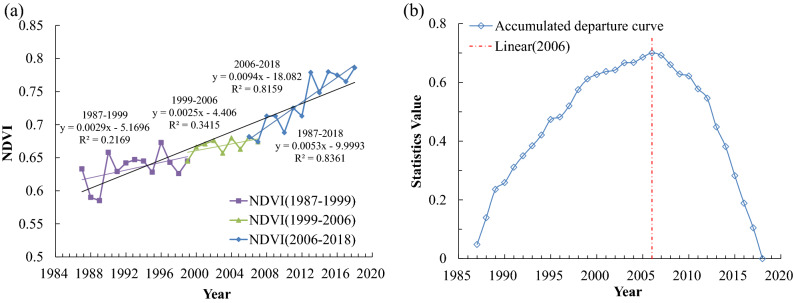
Change trend (**a**) and abrupt point analysis (**b**) of maximum NDVI in growing seasons in Qin Mountains (1987–2018). The change trend of maximum NDVI in growing seasons extracted from Google Earth Engine (GEE). The points that transformation from upward to downward has been considered as the abrupt change of data sequence, and the “Statistics Value” in the y-axis refer to the accumulated departure. Besides, considered the implementation of “Grain For Green Project” in 1999 in China, we divided the whole study time into three periods: 1987–1999, 1999–2006, 2006–2018.

The inter-annual NDVI is extracted from the mean of the pixel values of maximum NDVI during the growing season (March to November) from 1987 to 2018 in the Qin Mountains based on linear regression analysis method in GEE. The results show that the vegetation situation in the Qin Mountains is at a relatively high levels (mean value of NDVI is 0.68) and the NDVI shows an obvious increase with annual change rates of 0.0053/a (R^2^ = 0.8361, p < 0.01) over the past 32 years (Fig. 2a). The abrupt point is found in 2006 (Fig. 2b) and the change rates after this point increase up to 0.0094/a (R^2^ = 0.8159, p < 0.01) (2006–2018), which is higher than that in 1987–1999 (slope = 0.0029, R^2^ = 0.2169, p < 0.01) and 1999–2006 (slope = 0.0025, R^2^ = 0.3415, p < 0.01).

#### NDVI variation based on elevation division

**Figure 3:**
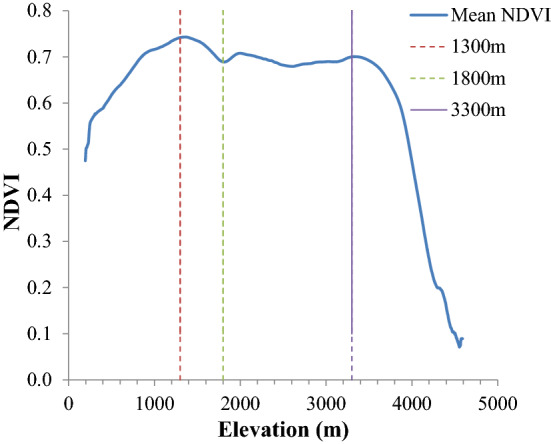
The NDVI change under different elevation in Qin Mountains (1987–2018). Statistics the NDVI value for every pixel in DEM image, and then used the Exponential Smoothing method in Excel to simplify the curve.

**Figure 4:**
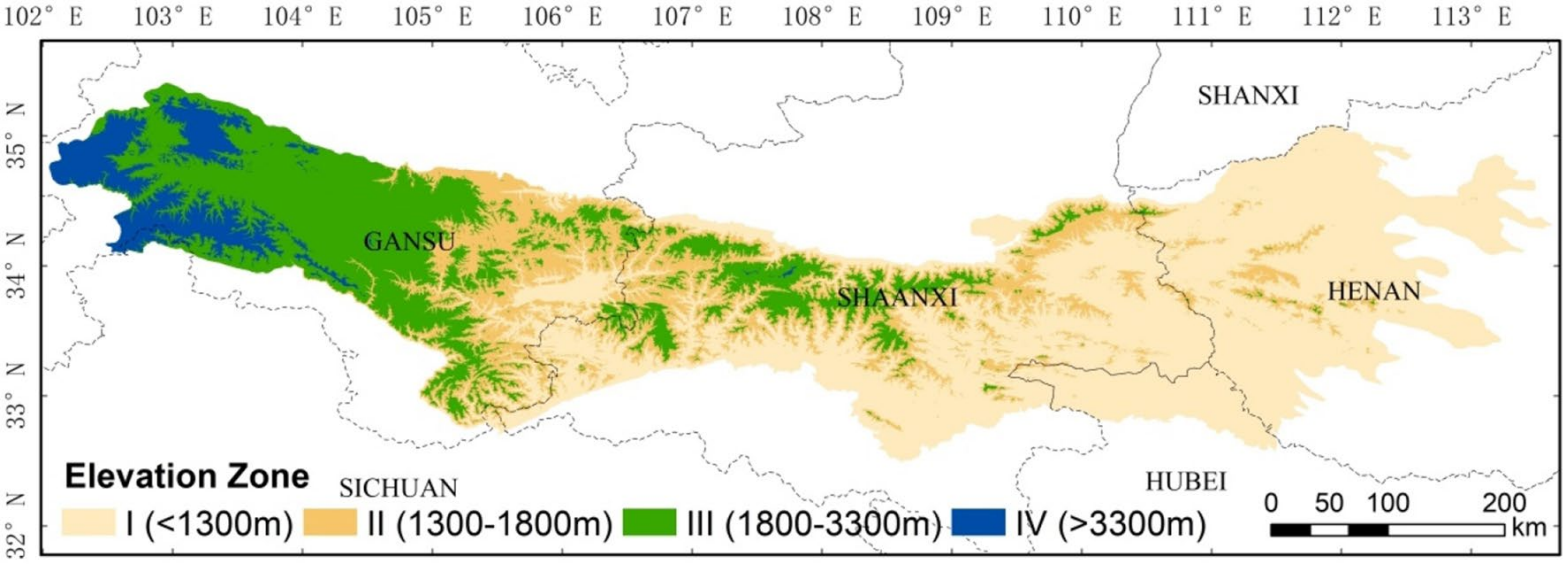
The spatial distribution of four Elevation Zone in Qin Mountains. According to the change rules of NDVI as the increased of elevation, we download DEM through GEE and divided the Qin Mountains into four Elevation Zones and mapping in the ArcGIS 10.2.

Previous studies divided the elevation according detail descriptions from “The outline of ecological environment protection of the Qin Mountains in Shaanxi Province”^13,14^ and Deng’s paper^10^ as following: (1) less than 1500 m (soil and water conservation district); (2) between 1500 and 2600 m (mixed forest of coniferous and broad-leaf and biodiversity area); (3) greater than 2600 m (coniferous forest shrub in meadow and biodiversity area). Considering vegetation cover obviously influenced by the different range of elevation we divided elevation in this paper according to the NDVI change rules with the increase of elevation, that are Zone I: < 1300 m, Zone II: 1300–1800, Zone III: 1800–3300 m, Zone IV: > 3300 m. Figure 3 displays the mean of maximum NDVI change in the growing season during 1987–2018 with the elevation increase. The results show that there is a noticeable increasing trend of NDVI at the elevation of less than 1300 m, which increased from 0.48 to 0.74. Then, NDVI started to decrease after reaching up to a peak value from 1300 to 1800 m. After that, the mean NDVI is stable at 0.7 between the 1800 and 3300 m and then decreased drastically above 3300 m. According to the NDVI change characteristics mentioned above and previous study researches, we divided the elevation into four Elevation Zones (I: < 1300 m, II: 1300–1800 m, III: 1800–3300 m, IV: > 3300 m) (Fig. 4) and statistics of the characteristics value of maximum NDVI at growing season for four Elevation Zones from 1987 to 2018 (Table 1). The results show that the NDVI increased continuously in all the elevation ranges from 1987 to 2018. The slope of NDVI in Elevation Zone I is 0.18, which is higher than any of other Elevation Zones. Besides, the change rate is especially higher in Elevation Zone IV (deviation is 0.0785).

**Table 1:** The characteristics of NDVI value under different elevation from 1987 to 2018 of Qin Mountains.

Elevation Zone	Elevation range (m)	Area (%)	NDVI				
			Mean	Slope	Maximum	Minimum	Std
I	< 1300	50.56	0.67	0.18	0.78	0.579	0.0568
II	1300–1800	20.20	0.71	0.15	0.816	0.605	0.0546
III	1800–3300	22.51	0.69	0.13	0.802	0.512	0.0597
IV	> 3300	5.72	0.65	0.12	0.785	0.481	0.0785

### NDVI changes in different vegetation type under various elevation zones

Further statistics for the mean NDVI and its change slope in different vegetation and at different elevation before and after abrupt point (2006) is shown in Table 2. It’s obvious that the mean NDVI and change slope all increased from period 1 to period 2. In detail, the areas below 3300 m (include Elevation Zone I, II and III) are mainly dominated by the cropland and forest. Forest vegetation type shows higher NDVI in the Qin Mountains, and it is more obvious on the Elevation Zone II and III (0.74 and 0.71 before 2006, 0.82 and 0.82 after 2006). The mean NDVI of Cropland on the Elevation Zone I are 0.55 and 0.66 during 1987–2006 and 2006–2018 separately, which is higher than that on other Elevation Zone. Although the grassland on these Elevation Zones account for small area, the mean NDVI changed obviously, especially on the Elevation Zone I and II during two periods. The forest and grassland occupy most of area on the Elevation Zone IV, which account for 35.8% and 62.7% of area in Elevation Zone IV. Compared with other Elevation Zones, the mean NDVI and slope for forest in this Elevation Zone shows low value both in period 1 and period 2. Moreover, the grassland here show a relatively higher NDVI value and higher increased rate than any other vegetation types in Elevation Zone IV, which reached up to 11.8%.

**Table 2:** Area percentage of each classification and mean NDVI as well as its slope for different vegetation types under four Elevation Zone during 1987–2006 and 2006–2018 in Qin Mountains.

Elevation zone	Vegetation type	Area (%)	Period 1		Period 2		Increased rate of mean NDVI (%)
			1987–2006		2006–2018		
			Mean NDVI	Slope	Mean NDVI	Slope	
I < 1300 m	Cropland	27.07	0.55	0.11	0.66	0.32	21.31
	Forest	62.24	0.67	0.11	0.79	0.29	17.00
	Grassland	8.23	0.56	0.13	0.67	0.36	19.46
II 1300–1800 m	Cropland	17.94	0.46	0.14	0.60	0.46	28.30
	Forest	78.85	0.74	0.07	0.82	0.24	10.55
	Grassland	2.87	0.57	0.10	0.66	0.42	16.17
III 1800–3300 m	Cropland	21.54	0.54	0.05	0.60	0.44	10.93
	Forest	70.17	0.71	0.04	0.82	0.27	16.09
	Grassland	7.60	0.63	0.11	0.66	0.29	4.45
IV > 3300 m	Cropland	1.43	0.57	0.06	0.62	0.28	7.72
	Forest	35.76	0.66	0.05	0.73	0.26	10.59
	Grassland	62.68	0.61	0.11	0.69	0.26	11.80

Area (%) refer to the area percentage of different elevation zone type.

### The relationship between NDVI and climate variables

#### The correlation coefficient between NDVI and climate variables in Qin Mountains

In time scale, the correlation coefficients between NDVI and climate variables show a continuous increasing trend (Table 3). Additionally, the correlation coefficients between NDVI and climate factors (precipitation, temperature and reference evapotranspiration) show obvious differences in the Qin Mountains over the past 13 years. The correlation coefficient between NDVI and precipitation is not very high, which increased from 0.06 to 0.21 over the past 13 years in Qin Mountains. The correlation coefficient between NDVI and thermal factors also show an increased trend, which reached up to the highest value in the year range of 1987–2018 and 1987–2016 for temperature and reference evapotranspiration, separately.

**Table 3:** Correlation coefficients between NDVI and climate factors at growing season over the past 13 years in Qin Mountains.

Year range	R_NDVI-P_	R_NDVI-T_	R_NDVI-PET_
1987–2006	0.06	0.1	0.16
1987–2007	0.06	0.11	0.17
1987–2008	0.05	0.13	0.17
1987–2009	0.05	0.14	0.18
1987–2010	0.08	0.14	0.18
1987–2011	0.12	0.15	0.2
1987–2012	0.11	0.16	0.21
1987–2013	0.13	0.24	0.28
1987–2014	0.13	0.22	0.26
1987–2015	0.14	0.22	0.27
1987–2016	0.17	0.27	0.29
1987–2017	0.18	0.29	0.28
1987–2018	0.21	0.31	0.24

R_NDVI-P_ R_NDVI-T,_ R_NDVI-PET_ refer to the correlation coefficient between NDVI and precipitation, between NDVI and temperature and between NDVI and reference evapotranspiration, separately.

#### Correlation coefficient under different elevation

**Figure 5:**
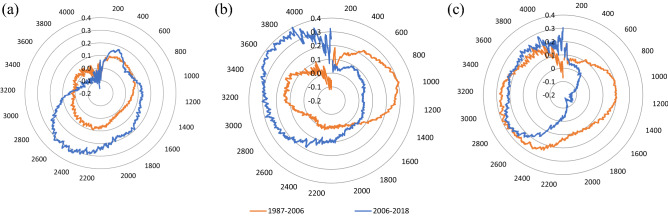
The correlation coefficient (**a**) between NDVI and precipitation, (**b**) between NDVI and temperature and (**c**) between NDVI and reference evapotranspiration climate factors under different elevation in Qin Mountains. The correlation coefficient between NDVI and hydro-climatic factors evaluated based on the pearson correlation method, which also available in the Google Earth Engine.

Further statistics for the NDVI value distribution following elevation change in Qin Mountains can be seen in Fig. 5. It is illustrated that the precipitation shows no obvious correlation or negative correlation with NDVI before 2006. The relatively high correlation between NDVI and precipitation occurred between elevations of 2300–2700 m. After that, the coefficient decreased rapidly and decreased from 0.1 to − 0.1 with the elevation higher than 3000 m. The high correlation coefficient between NDVI and temperature is mainly distributed in 0–1300 m before 2006, but after 2006, the high coefficient is in the elevation higher than 3000 m. The relatively high correlation coefficient between NDVI and reference evapotranspiration occurs in elevation between 800–1200 and 2400–3300 m before 2006, and the higher coefficient just existed in the elevation that higher than 2600 m after 2006.

## Discussion

The Qin Mountains region is one of the most important climatic boundaries that divide North and South of China with complex geological structure^15^, its vegetation covers changed significantly over the past decades. From 1950s, the Chinese government developed a series of afforestation activities in China in order to effective improve the treatment and extent the implement scale^16,17^. The “Green for Grain Project” (GFGP) played a significant role especially in areas such as the Qin Mountains that have abundant species and complex ecosystems^18,19^. The results shown in this study indicated that NDVI increased continuously and significantly in most area of the Qin Mountains over the past 30 years (R^2^ = 0.84, p < 0.01), which is consistent with the conclusions of the vegetation coverage that apparent improvement were made by other studies in China^20^. Additionally, the abrupt point detected by Accumulated Departure method in our research was found between 2006, which is closer to the conclusions made by other studies. For example, Cao^21^ pointed out that more than half area of the Loess Plateau shows a significantly increased trend during 2000–2013 and the change point generally occurred in the years of 2007–2010. The results presented by Deng^10^ show that fraction vegetation cover in the narrow Qin Mountains region started increasing from 2000 and suddenly raised around 2005. Those results indicated that the vegetation cover did not improve immediately after afforestation activities but increased a few years later.

Besides, some researchers also point out that the NDVI is not only influenced by climate factors but also impacted by human activities such as positive and negative activities^22–24^. As the development of afforestation policy, the correlation coefficient between vegetation growth and climate decreased^25^ especially in the low elevation areas which are located in the key region of soil and water conservation implementation, such as Elevation Zone I (below 1300 m) of the Qin Mountains that is dominated by the cropland and forest. The NDVI was mainly influenced by the thermal factors before 2006, and the impact is then weakened after 2006 due to the positive activities such as afforestation, terrain construction. Additionally, a previous study indicated that precipitation was the dominant factor in the area of coniferous forests and grassland^26^, which is consistent with the results in our study, that is, the correlation coefficient between NDVI and precipitation in the elevation between 2200 and 2600 m that distributed by the mixed forest of coniferous and broadleaf, shows a relative high value. However, NDVI on the elevation areas higher than 3300 m which is dominated by the grassland and forest still obviously influenced by thermal factors rather than human activities after 2006.

**Figure 6:**
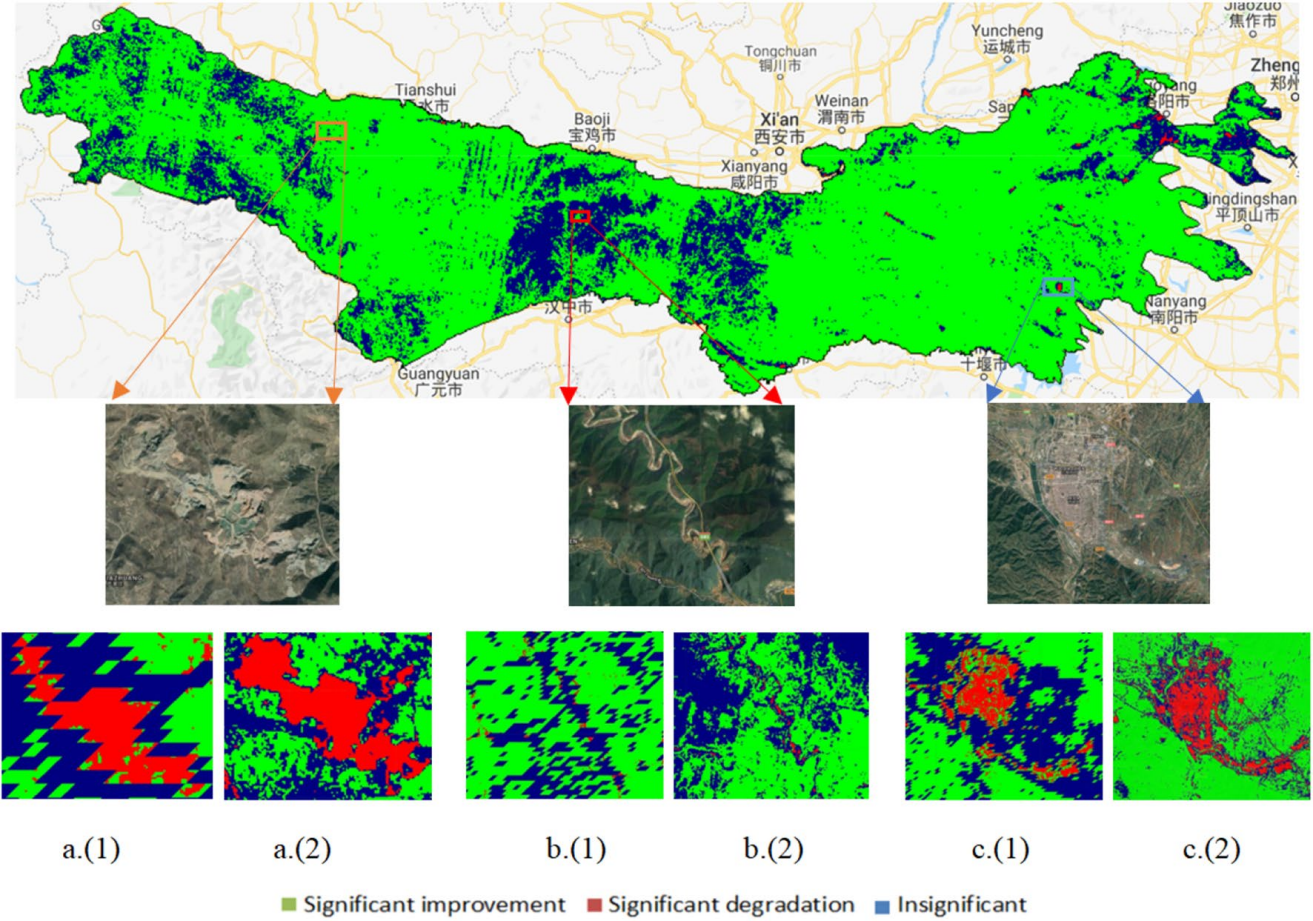
The significant change of NDVI from 2000 to 2018 in Qin Mountains. Numbers (1) and (2) refer to the NDVI derived from the MODIS and Landsat dataset based on the GEE (https://earthengine.google.com/), respectively. Considering to compare the difference between MODIS and Landsat, we calculated significant change of NDVI based on the two datasets by using same method (Theil–Sen Trend). Meanwhile, we choose the area with vegetation degradation such as residential, transport area, and mining area to further compare this difference.

The most important thing in the study of vegetation change is monitoring the location and area change due to degradation^27^ such as industrialization and urbanization which can change the vegetation growth situation to a certain extent^28^. For example, the mining area, reservoir (Fig. 6a is a typical mining area in the middle of Gansu Province), road construction (a mountain road in the western of Shannxi which showed in the Fig. 6b) and cities and counties such as Gannan, Jone, Minxian, Tianshui of Gansu Province; Taibai, Ankang, Shangluo of Shaanxi Province and Xixia, Sanmenxia, Mianchi, Dengfeng of Henan Province (Fig. 6c is an example for Xixia in Henan Province) distinguish the location of degradation area using remote sensing datasets. Besides, the higher resolution images can not only be used for extracting the more real situation of vegetation information^29,30^, but also for monitoring the area of land cover change such as urbanization and afforestation^31,32^. In the previous studies such as Du et.al and Zhao et.al, the GIMMS and MODIS NDVI datasets are considered as the best available datasets for long-term NDVI trend analysis, respectively^2,8^. However, they have their own disadvantages in term of resolution and time series. The Landsat dataset and the Google Earth Engine (GEE) provide a valuable opportunity to obtain a more detailed and long-term surface information and make it possible to monitor the vegetation cover changed significantly in the past several decades^12,33^. It is clear that the area of significant degradation extracted by the Landsat dataset has clearer bounds, and can reflect more internal information than using MODIS-derived NDVI dataset (Fig. 6).

The NDVI spatial distribution as well as the change of vegetation cover and its correlation between climate variables and vegetation index are all influenced by the elevation. At an elevation of less than 1300 m, NDVI in the Qin Mountains showed a significant increasing trend as the elevation increased. Meanwhile, the NDVI increased was obviously during the periods from 1987 to 2018. This was mainly due to the influence of positive effects of human activities (such as reforestation after “Grain For Green”). After that, the mean NDVI decreased drastically above the elevation of 3300 m where significantly related to climate change, which is consistent with previous study that thought the high altitude plants (e.g. *Abies fargesii*) suffered a large number of deaths due to habitat changes as the climate warming^34^.

Additionally, the vegetation cover is influenced by a lots of climate factors not only precipitation, temperature and ET chosen from this paper. Other factors like soil moisture, humidity, aridity index are also the important variables influenced NDVI at different degree, and those factors show lag effect with NDVI^35^. So, in the future, we will look further into analyzing the relationship between NDVI and climate variables under the seasonal scale^9^ and considerate the lag effect between NDVI and climate variables.

## Materials

### Study area

**Figure 7:**
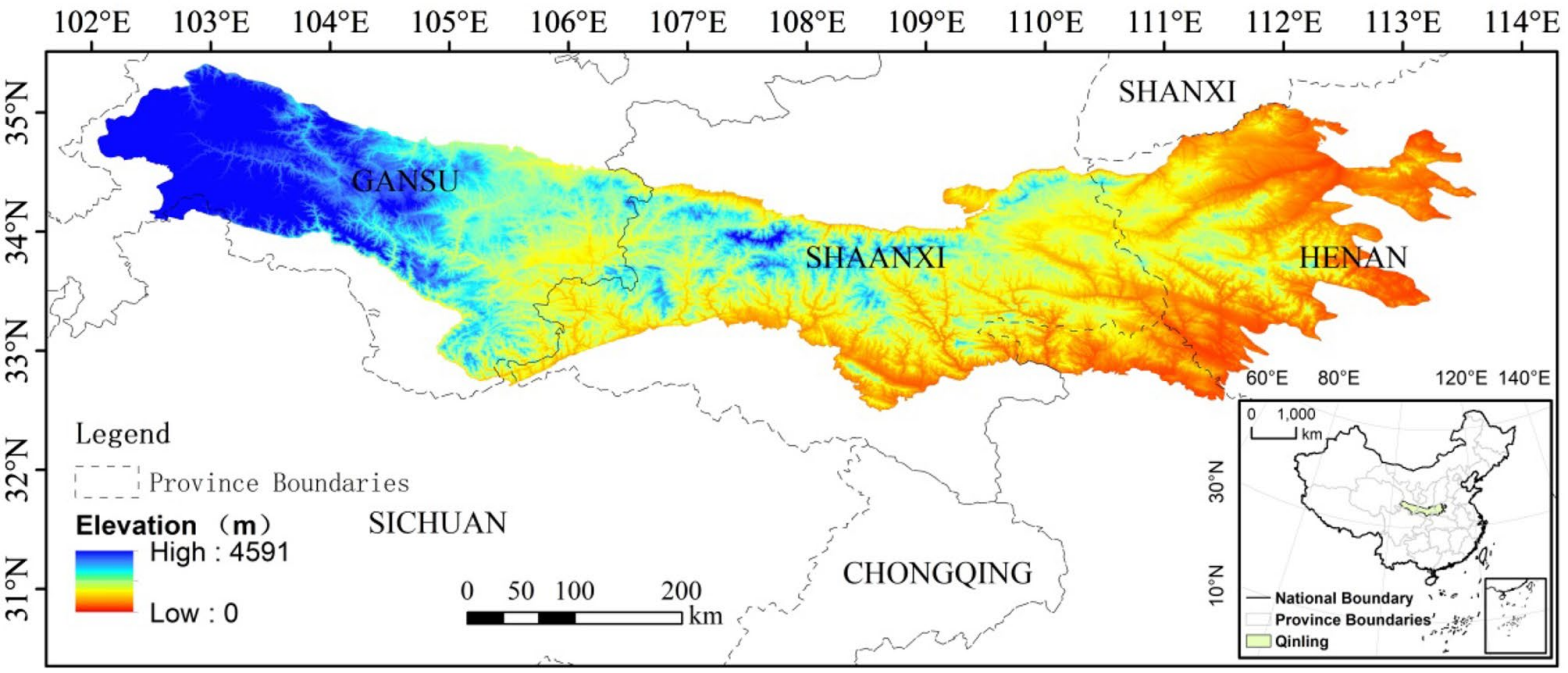
Geographic location of Qin Mountains. This Figure include the Boundaties of Chinese Province and Qin Mountains, and the Elevation as the base map. Besides, the location of Qin Mountains in China has been show in the right bottom of this Figure. The Digit Elevation Model (DEM) obtained from the GEE.

The Qin Mountains are the major east–west mountains located in southern Shaanxi Province, China (104° 30′–110° 05′ E, 32° 40′–34° 35′ N) (Fig. 7). The covered area of the Qin Mountains is 15.59 km^2^ and the elevation is ranging from 95 to 4591 m^36^. The mountains provide a natural boundary of North and South China and support a huge variety of plants and wildlife. The climate on the north side is a warm and sub-humid area and precipitation is often below 750 mm. The land cover dominated by the cropland and temperate deciduous broad-leaved forest. The climate on the south side is much more humid and hotter. It is dominated by the subtropical humid climate and subtropical evergreen broadleaved forest with fertile soil^37^. The plants distribution varies with the altitude change, for example, Quercus variabilis forest (1200–1400 m on the northern and southern slopes), Q. aliena var. acuteserrata forest (1200–1650 m versus 1400–2050 m), Q. mongolica forest (1650–2300 m only on the northern slope), and Betula albo-sinensis forest (2300–2600 m versus 2250–2500 m)^36^.

## Dataset

### Satellite imagery

**Figure 8:**
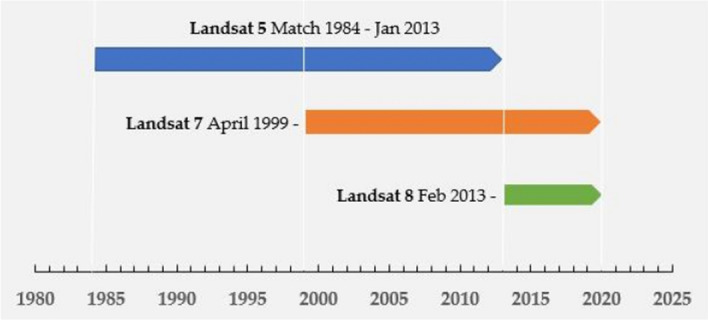
Available Landsat images from Landsat surface reflectance products. The Landsat datasets such as Landsat 5, Landsat 7 and Landsat 8, which produced by different sensors. Although they have same resolution, the time series are difference.

The satellite imageries used in this paper were obtained from Landsat surface reflectance products which is atmospherically corrected surface reflectance from the Landsat 5 ETM and Landsat 7 ETM + (processed by Landsat Ecosystem Disturbance Adaptive Processing System (LEDAPS) algorithm) and from Landsat 8 OLI/TIRS sensors (processed by using Landsat Surface Reflectance Code (LaSRC) algorithm) (Fig. 8). These images contain the pixel quality attributes such as cloud, shadow, water and snow mask generated by the Function of Mask (CFMASK) algorithm which can help user to select the appropriate images according to different requirement. More importantly, the Landsat derived have 30 m resolution, which is ideally images suited for local or regional scale research^7^. This kind of images can be access in the Earth Engine Data Catalog (https://developers.google.com/earth-engine/datasets) and process through Google Earth Engine. In our study, we choose the Landsat 5 from 1987 to 2011, Landsat 7 in 2012, and Landsat 8 from 2013 to 2018 as the basic data. Among these data, we process Landsat 7 through the gap-filling method.

### Digital elevation model

The Shuttle Radar Topography Mission (SRTM) digital elevation used in this paper is from SRTM V3 product (SRTM Plus), which was provided by NASA JPL at a resolution of 1 arc-second (approximately 30 m)^38^. We derived the elevation data based on the Google Earth Engine (Fig. 7).

### Land cover data

**Figure 9:**
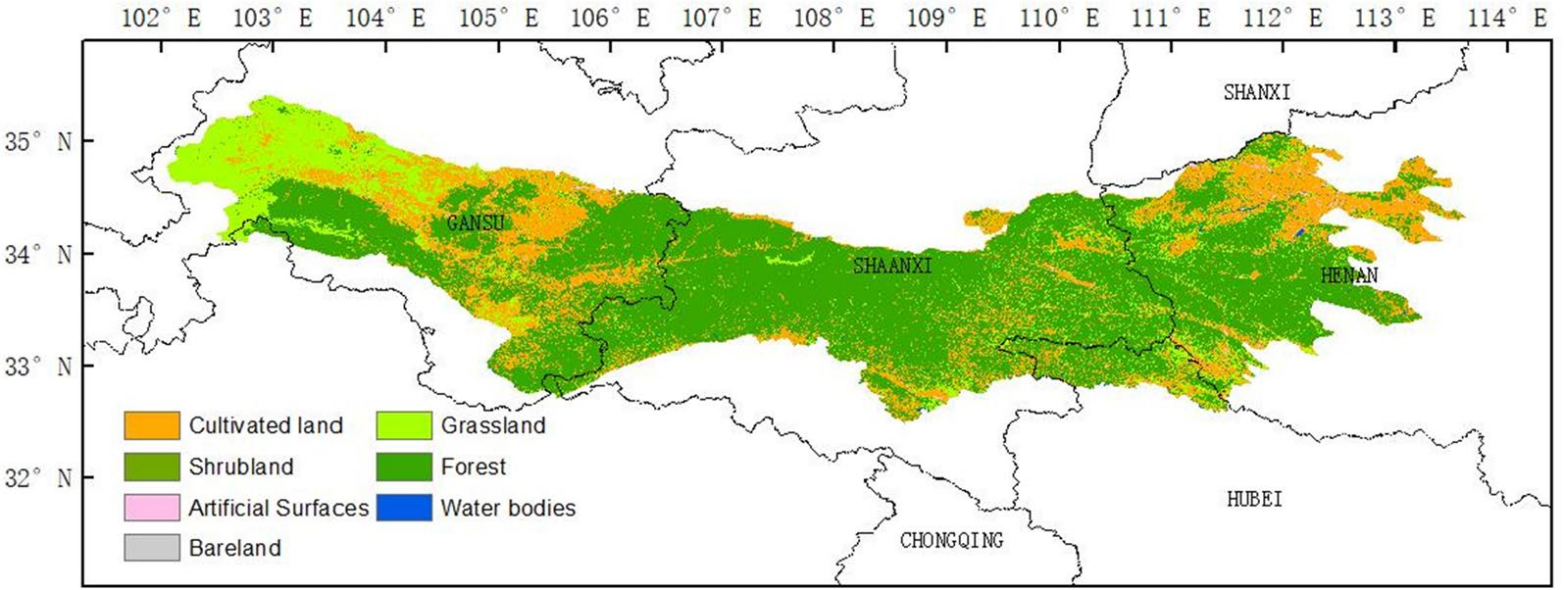
The spatial distributions of Land cover in the Qin Mountains. The distribution of vegetation type in this study is obtained from the Global Land Cover Map with a spatial resolution of approximately 30 m (GlobeLand30) in 2010 (www.globeland30.org), which obtained from the GEE. The vegetation types in the Qin Mountains were grouped into cultivated land, forest, grassland, shrub land, water bodies (include wetland), artificial surfaces and bare land. Their area percentage account for 22.53%, 65.85%, 10.12%, 0.12%, 0.38%, 0.99%, 0.005%, respectively.

The distribution of vegetation type in this study is obtained from the Global Land Cover Map with a spatial resolution of approximately 30 m (GlobeLand30) in 2010 (www.globeland30.org)^39^. The vegetation types in the Qin Mountains were grouped into cultivated land, forest, grassland, shrub land, water bodies (include wetland), artificial surfaces and bare land. Their area percentage account for 22.53%, 65.85%, 10.12%, 0.12%, 0.38%, 0.99%, 0.01%, respectively (Fig. 9).

### Climate data

Climate dataset used in this study is Monthly Climate and Climatic Water Balance for Global Terrestrial Surfaces (TerraClimate) which available in the GEE. This dataset combines the high-spatial resolution WorldClim dataset with time-varying data from CRU Ts4.0 and the Japanese 55-year Reanalysis (JRA55). It is interpolated time-varying anomalies from CRU Ts4.0/JRA55 to the high-spatial resolution climatology of WorldClim to create a monthly high-spatial resolution dataset (about 5 km) with broader temporal record^40^. As the key elements influencing regional water balance, water cycle and land surface energy flow, the climatic variables such as mean value of precipitation, temperature, reference evapotranspiration (PET, ASCE Penman-Montieth) in the growing season (March to November)^41^ from 1987 to 2018 are considered in this paper^42,43^.

## Methods

In order to derive the higher resolution images, we proceeded and analyze the Landsat surface reflectance dataset based on the Google Earth Engine (GEE) and use the Accumulated variance analysis and Theil–Sen Trend method to detect the NDVI change trend as well as abrupt point. Meanwhile, the Pearson correlation method is applied to analyze the relationship between NDVI and climate variables.

### Satellites image processing

For this study, we are processed Landsat scenes based on the GEE as following steps: (i) removing the cloud and shadow pixels according to the pixel data quality of flag information; (ii) calculating NDVI for each image according to following equation:

1 $$\mathrm{NDVI}=(NIR- RED)/(NIR+ RED)$$

where NIR, RED refer to the near infrared band (band 4—Landsat 5, 7; band 5—Landsat 8) and red band (band 3—Landsat 5, 7; band 4—Landsat 8); and (iii) combining all Landsat-derived images and extracting NDVI according to study destination (study area and study time series). In order to reflect vegetation cover at the optimum level, the maximum NDVI in growing season (March–November) was extracted in this paper 2.

### Trend analysis

To identify the abrupt point for the whole research period and detect trends of NDVI and climate factors, the Accumulated variance analysis and Theil–Sen Trend method are applied. Accumulated variance analysis is a test method based on mean value, which can conduce to detect the changes and abrupt of data sequence by observing the residual mass curve. In general, the points that transformation from upward to downward has been considered as the change point of data sequence^44^. We use this method to detect the change point and divide whole study time series into different periods to further compare the vegetation change before and after vegetation restoration. To analyze the trends of NDVI, Theil-Sen Trend method are applied, which are all available in the Google Earth Engine.

According to methods mentioned above, we divide the NDVI change into three categories according to different value of slope and p-value as following: (i) Significant greening (slope > 0, p-value < 0.05); (ii) Insignificant (slope = 0, slope > 0, slope < 0, p-value > 0.05); and (iii) Significant degradation (slope < 0, p-value < 0.05) to assess the significant level of vegetation change.

### Causality analysis

Additionally, in order to find out the main drivers of vegetation cover variability, the Pearson correlation method is applied again to construct the relationship between NDVI and climate variables in each pixel. And the rules for judging the positive, negative significant, and insignificant correlations between NDVI and climatic variables are set as follows: (i) significant positive correlation (coefficient > 0, p-value < 0.05); (ii) insignificant (coefficient = 0, coefficient > 0, coefficient < 0, p-value > 0.05); and (iii) significant negative correlation (coefficient < 0, p-value < 0.05).

## Conclusion

This study investigated the NDVI distribution and dynamics under the high-resolution images, and the relationship between related climate variables under the different gradient of the Qin Mountains from 1987 to 2018. The implementation of “Grain For Grain” policy in China lead to the vegetation greening obvious in the Qin Mountains, especially after 2006. The increase in NDVI during 2006–2018 was more than 3 times than that in 1987–1999 and 1999–2006. Moreover, most of the Qin Mountains are dominated by the NDVI value larger than 0.5, which are reached reaching up to 92.2% of total area. The rest of place such as residential or mining area, the NDVI show the lower value and decreased trend. Besides, the NDVI show a general trend that increased then decreased as altitude increases. In time, the NDVI in the elevation less than 3300 m (include Elevation Zone I, II and III) increased more apparent than high elevation especially in the area covered by cropland and grassland. The area with an elevation high than 3300 m (Elevation Zone IV), where mainly covered by forest, the NDVI show a lower value. Before 2006, the NDVI in areas with an elevation below 3300 m was mainly influenced by the temperature and reference evapotranspiration, but interference by human activities after 2006. However, the NDVI increase in the area above 3300 m was obviously influenced by the temperature and reference evapotranspiration all the time. Our research pays more attention to the long-time series and high resolution NDVI distribution and trend under different elevation which may helpful for detect the vegetation dynamics in detail and can help to implement relevant policies to relieve the vegetation degradation.
